# Metabolic Functions of Lysine 2-Hydroxyisobutyrylation

**DOI:** 10.7759/cureus.9651

**Published:** 2020-08-11

**Authors:** Shaoying Huang, Donge Tang, Yong Dai

**Affiliations:** 1 Department of Clinical Medical Research Center, Shenzhen People's Hospital, Shenzhen, CHN; 2 Department of Clinical Medical Research Center, Shenzhen People’s Hospital, Shenzhen, CHN

**Keywords:** lysine 2-hydroxyisobutyrylation, post-translational modifications, metabolism

## Abstract

Lysine 2-hydroxyisobutyrylation (Khib) is one of the newly identified post-translational modifications (PTMs) primitively identified by mass spectrometry (MS). Research in animals suggests that histone Khib is related to regulation of chromatin and cellular functions. However, there is no review to summarize and elaborate on the current understanding of Khib. In this review, we start with the basic description of Khib, such as sequence preference and subcellular localization. We then analyze the lysine 2-hydroxyisobutyrylated sites with the purpose of learning its functions and activities. We finally discuss the regulation toward Khib and the way it regulates, thus opening an avenue for us to illustrate the diverse cellular metabolites associated with (Khib).

## Introduction and background

Protein post-translational modifications (PTMs) are important epigenetic modifications of the nucleosomal core histones that occur during the orchestration of chromatin dynamics [1]. PTMs can alter a protein’s properties through covalent linkage with a target for proteolytic cleavage or the modification or addition of an amino acid functional group, thereby fine-tuning its function [2]. Prediction of DNA coding capacities suggests that the number of PTMs substantially exceeds the number of proteins, because enzymes dedicated to such protein modifications include hundreds of human protein kinases, protein phosphatases and proteases [3]. Different types of PTMs are being discovered, which influence the properties of proteins, such as their interaction, cellular localization, enzymatic activity and stability [4-8], and regulate biological activities, including interactions between proteins, protein degradation, subcellular localization, gene transcription, signal transduction and conformational change [9-11].

Several new types of lysine acylations have been discovered over the past decade. Lysine acylations compose a family of PTMs of histones that affect gene expression and include propionylation, β-hydroxybutyrylation, crotonylation, butyrylation, malonylation, glutarylation and 2-hydroxyisobutyrylation; these modifications differ in function and structure from the widely studied histone Lys acetylation. Although there is a wide range of PTMs, this review focuses on Khib.

Hydroxyisobutyrate (Hib) is a short-chain fatty acid that occurs in a vast variety of biofluids in humans, including blood, urine and feces, at micromolar concentrations, and its occurrence at high levels in the urine of obese patients is associated with the abundances of specific taxa of gut microbiota [12-16]. Hib mediates Khib, a widespread histone marker, and is a precursor for the synthesis of 2-hydroxyisobutyryl-coenzyme A (CoA) [12].

Khib is a newly identified PTM and was first found in histone proteins from human and mouse. It is known to be an evolutionarily conserved PTM and exhibits a unique genomic distribution with a unique chemical structure. Its characteristics distinguish it from the well-studied histone lysine methylation (Kme) and lysine acetylation (Kac) [12]. This newly identified chemical modification occurs in a variety of organisms and plays roles in the biosynthesis and degradation of proteins and energy metabolism [13].

In this review, we discuss the properties of this novel modification and the relationship between Khib and metabolic processes. We first describe the discoveries at Khib sites, including the sequence preference and subcellular localization of this acylation and its domains and motifs. Through the analysis of lysine 2-hydroxyisobutyrylated sites, we illustrate the functions of the 2-hydroxyisobutyrylated proteins and enzymes, providing insight into the ways Khib influences metabolism. We then address the factors that can regulate Khib.

## Review

Khib properties

Khib substrates have multiple modification sites. For example, hundreds of proteins can be modified by Khib in HeLa cells at only one site, whereas 80 proteins have more than 10 sites [13]. The negatively charged amino acids are enriched at both −1 and +1 positions, and the positively charged amino acid lysine is enriched at positions −6, −5, +5, and +6 [13]. In addition, at the −1 and +1 positions, arginine residues are underrepresented with lysine, and proline is depleted at a majority of these positions, which is assumed to be distinct from the flanking sequence preferences reported for lysine succinylation (Ksucc), lysine malonylation (Kmal), and Kac [14-17].

PTMs, such as Ksucc and Kmal, are markedly enriched in mitochondria [15-18]. However, only a small minority of Khib proteins have been annotated in mitochondria, whereas the majority of Khib proteins have been localized exclusively or partially in nucleus and cytosol. This observation suggests that the regulatory mechanism underlying Khib modification differs from that of Ksucc and Kmal, whereas the Kac substrates are similar to the Khib substrates in subcellular distribution [13]. For Kac, most of the substrates usually reside in the nucleus or cytoplasm, but approximately 5% or less of proteins acetylated by lysine are in the mitochondria [19]. According to a study of cellular components, 10% of the modified proteins are in the membrane, and less than 10% are in the ribosomal subunit, with the majority of modified proteins being in the cytosol [20]. The above observations clearly suggest that Khib may regulate intracellular processes and cell structure and major functions of the Khib pathway are likely widespread in diverse subcellular compartments.

Analyses of the structural properties of Khib-modified proteins in* Proteus mirabilis* have revealed that the major domains in Khib-modified proteins are P-loop domains containing nucleic acid-binding, nucleoside triphosphate hydrolase, aldolase-type TIM barrel, and Rossmann-like α/β/α sandwich fold domains. Alanine was found to be overrepresented at +1, +2, -1, and -3 positions in *Proteus mirabilis *in a frequency analysis of position-specific amino acids from the surrounding lines of a sequence of 2-hydroxyisobutyrylated lysine residues [20]. Khib and the majority of known histones, Kac and Kcr, exist at the N-termini. However, Khib also occurs in their main globular domains. The modified lysine combines with other molecules by hydrogen bonds via a hydroxyl group, which is of great importance for the functional regulation of Khib-modified protein [21].

Analysis of Khib sites

In *Proteus mirabilis,* Khib has been observed to occur at major functional regions of Khib-modified proteins, near other PTMs. For example, eight Khib sites occur in the NAD-binding region of glyceraldehyde-3-phosphate dehydrogenase, suggesting that Khib modification might play a role in regulating events at specific positions and possesses potential biological functions [20].

Khib has also been observed to share the same lysine sites as other modifications in *Proteus mirabilis*. For example, Khib shares seven lysine sites with Kac. In glyceraldehyde-3-phosphate dehydrogenase (GAPDH), the same 10 sites are modified by both Ksucc and Khib, which suggests that different PTMs may undergo functional cross-talk and potentially compete with each other at specific lysine sites [20].

Functional annotation of 2-hydroxyisobutyrylated proteins and enzymes

Most of the 2-hydroxyisobutyrylated proteins in *Proteus mirabilis* that have been identified via KEGG pathway analysis are enriched by the translation, proteolysis, cell division, and tricarboxylic acid cycle pathways and, according to the Gene Ontology (GO) functional classification, are related to metabolism and various binding targets [20].

Glycolysis converts glucose into pyruvate through 10 enzymatic steps [22]. Five out of the 10 key enzymes are heavily modified by Khib: phosphoglycerate kinase 1, pyruvate kinase isoform M2, glyceraldehyde-3-phosphate dehydrogenase, fructose-bisphosphate aldolase A, and alpha-enolase (ENO). ENO coverts 2-phosphoglycerate to the phosphoenolpyruvate (PEP) as a high-energy intermediate, and K343 is 2-hydroxyisobutyrylated as its activity center residue. This modification disrupts the correlation of the active-site residues and hampers enzymatic activity, where Khib is likely to disrupt the binding interaction, thus affecting protein function [13].

The top enriched protein complexes in both HeLa cells and *Proteus mirabilis *are associated with ribosome, proteasome, and spliceosome [13,20]. In addition, in *Proteus mirabilis*, significant enrichment of Khib has been identified in the CCT micro-complex, the H2AX complex, the TNF-α/NF-κB signal transduction pathway, and the DNA-PK-Ku-eIF2-NF90-NF45 complex [20]. The TNF-α/NF-κB signal transduction pathway is involved in various biological processes, and nine out of 14 subunits in this complex are 2-hydroxyisobutyrylated [13]. The DNA-PK-Ku-eIF2-NF90-NF45 complex plays a pivotal role in DNA double-strand break repair, and seven of the eight proteins in this complex are 2-hydroxyisobutyrylated [13-22]. These results suggest that Khib is involved in diverse cellular functions, including protein synthesis and degradation, cellular signaling, and DNA repair. The interaction networks and their correlations to 2-hydroxyisobutyrylated proteins are similar to those of acetylated proteins, suggesting that Khib has functions similar to acylation in biological processes.

In the HeLa Khib proteome, Khib is enriched in macromolecule transport-related pathways, such as RNA transport and protein export pathways; energy metabolic networks, such as fatty acid metabolism; the citric acid cycle and pyruvate metabolism; and the ribosome, spliceosome, and proteasome pathways [13]. A large proportion of metabolic enzymes are 2-hydroxyisobutyrylated in biological processes. For example, in the pentose phosphate pathway, TCA cycle, and glycolysis/gluconeogenesis, 54 crucial enzymes are 2-hydroxyisobutyrylated, and 26% of these enzymes have more than 10 2-hydroxyisobutyrlated sites [13].

Khib regulation of metabolism

Recent studies have demonstrated that lysine modifications suppress enzymatic activity, such as succinylation and malonylation [23-24]. The observation of Khib on metabolic enzymes leads us to speculate whether Khib also acts to suppress enzymatic activity.

The substrate 2PG binds to two different substrate-binding pockets in an identified Khib site (K343) of ENO prior to modification. As a result, the Khib (K343) may destroy the binding environment such that the substrate cannot adequately fit in the binding site, affecting the enzymatic activity of ENO. Thus, Khib (K343) is a negative factor of modification during the regulation of ENO, and molecular docking results indicate that Khib might affect catalytic activity through the isolation of the substrate and the active sites as a regulatory mechanism of ENO as well as its substrate 2PG [20].

Regulation of Khib

Khib can be regulated by carbon sources; considering that the corresponding acyl-CoAs of Khib are likely the acyl donors, 2-hydroxyisobutyryl-CoA (HibCoA) likely generates Khib as a cofactor [23]. HibCoA is a building block in bacteria for industrial polymer synthesis, and it can be regulated by carbon sources [25]. On the other hand, several 2-hydroxyisobutyrylated proteins are extensively involved in metabolic pathways, such as the pentose phosphate pathway and glycolysis/gluconeogenesis, indicating that such modification may significantly influence metabolism. These observations raise some interesting questions. Are these modifications regulated by carbon sources? Observations of the ways of different carbon sources influencing 2-hydroxyisobutyrylation status suggest that carbon sources can influence the emergence of Khib, and the carbon sources of glucose, pyruvate, and 2-hydroxybutyrate (2-HIBA) can increase 2-hydroxyisobutyrylation [20].

Regarding the differences in regulation among these three carbon sources, further investigations of HibCoA in metabolic pathways in *Proteus mirabilis *indicate that 2-HIBA may generate HibCoA directly [20]. Since pyruvate and glucose need several additional steps to generate HibCoA, they can be utilized by metabolic bypasses, indicating that the different positions of carbon sources may determine the metabolic regulation capacity of Khib in *Proteus mirabilis* and that Khib may mediate adaptation to various carbon sources. Therefore, the modification may be associated with metabolic regulation.

A recent report on* Saccharomyces cerevisiae *has shown that conditions of reduced glucose lead to diminished modification, whereas glucose and fructose can restore the H4K8hib level promptly after glucose starvation [26]. In contrast, galactose fails to rescue H4K8hib as a fermentable but “secondary” carbon source, suggesting that it requires a preferred fermentable sugar. The fast regeneration of H4K8hib modification depends on the glycolysis pathway after glucose starvation. Taken together, these observations suggest that H4K8hib is strictly regulated by glucose/fructose availability and establish a connection between Khib and carbon metabolism.

By the same enzyme from other lysine acylations: In addition to catalyzing acetylation or deacetylation, some lysine acetyltransferases and histone deacetylases have been reported to catalyze additional acylation or deacylation reactions [27-30], suggesting that the regulation and function of these new acylations may be similar or identical to those of histone acetylation [26]. Histone deacetylases can remove acetyl groups as an enzyme at the epsilon position of lysine side chains [30-32]. As a member of lysine acetyltransferases, p300 has enzymatic activity not only for lysine acetylation but also for lysine butyrylation, propionylation, and crotonylation [27-28]. In mammalian cells, histone deacetylase 2 (HDAC2) and HDAC3 can serve as “erasers” to remove Khib and exhibit the highest activity for de-2-hydroxyisobutyrylation [13]. Knockdown of HDAC2 or HDAC3 increases the global level of histone Khib, whereas expression of HDAC2 or HDAC3 reduces the global level of histone Khib. Therefore, some lysine acetyltransferases and histone deacetylases may have activity toward Khib, and multiple acylations, including Khib, can possibly be catalyzed by the same enzyme.

Khib regulates glycolysis

Glucose is a major carbon source in most cells of the human body and participates in energy production and biosynthesis, which are of crucial importance for cell proliferation, growth, and survival. Strong regulation of systemic glucose homeostasis in response to cues, such as hormones, nutrients, and environmental cues, is critical for survival [33-35].

Glycolysis is a cascade of 10 pivotal enzyme-catalyzed reactions. Remarkably, five of the 10 glycolytic enzymes are 2-hydroxyisobutyrylated by EP300: glucose-6-phosphate isomerase (GPI), 6-phosphofructokinase muscle (PFKM) of the ATP-dependent type, ALDOA, PGK1, and alpha/gamma-enolase (ENO1/2) [36].

EP300, a transcriptional coactivator, regulates gene transcription via histone acylation [37], having the capacity to catalyze various types of lysine acylations [28]. In several different human cell lines in vitro, EP300 has been shown to serve not only as a histone acetyltransferase but also as a histone 2-hydroxyisobutyryl transferase, and it responds to nutritional cues as a lysine 2-c, thereby regulating glycolysis [36]. EP300-mediated Khib is selectively enriched in pathways, such as carbon metabolism, amino acid synthesis, and glycolysis or gluconeogenesis [36], suggesting that EP300 may modulate the homeostasis of cellular metabolism through Khib. Therefore, EP300 deficiency is expected to impair glycolysis. Furthermore, knockdown of EP300 significantly diminishes Khib levels on several EP300-dependent Khib-specific sites of key glycolytic enzymes, including ENO1, decreasing their catalytic activities. Collectively, these observations suggest that EP300 mediates nutritional regulation of cell survival through 2-hydroxyisobutyrylation of glycolytic enzymes in various human cells [36].

## Conclusions

New types of modifications continue to be discovered on both histones and non-histones in various cellular metabolic processes. Notably, 50% to 90% of the proteins in the human body need to be modified after translation. This modification determines the physical and chemical properties of the proteins, including their primary and tertiary structures, and their biological functions (such as enzyme activity). In this review, we discuss the sequence and domain preferences of Khib and the amino-acid residues enriched at Khib sites; these properties make the sites of Khib distinct from those of the other acylations to some extent. The subcellular distribution and regions of Khib inspire us to question the functions it has and roles it potentially plays in multiple cellular processes. The identification of 2-hydroxyisobutyrylated proteins and enzymes provides insight into the enzyme activities concerned with Khib. Such investigations will enhance our understanding of the associations of Khib with human diseases and aid the development of new methods for early diagnosis and treatment.
